# Galveston Medical Club

**Published:** 1886-02

**Authors:** 


### GALVESTON MEDICAL CLUB.

This organization, composed of the leading members of the profession in the Island City, held its annual election of officers for 1886 on January 4th ult., with the following result:

President, Dr. C. H. Wilkinson; Vice-Presidents, Drs. E. Goldman and A. Pope; Secretary, Dr. Wm. E. Fisher; Treasurer, Dr. I. Landergen; Executive Committee, Drs. A. W. Fly, H. P. Cook and H. West.

